# Electro-mediated drug administration of mitomycin C in preventing non-muscle-invasive bladder cancer recurrence and progression after transurethral resection of the bladder tumour in intermediate- and high-risk patients

**DOI:** 10.1080/2090598X.2020.1816150

**Published:** 2020-08-31

**Authors:** Roberto Carando, Emiliano Soldini, Simone Cotrufo, Michele Zazzara, Giuseppe M. Ludovico

**Affiliations:** a Klinik für Urologie, Luzerner Kantonsspital, Luzern, Switzerland; b Clinica Luganese Moncucco, Lugano, Switzerland; c Clinica S. Anna, Sorengo, Switzerland; d Clinica S. Chiara, Locarno, Switzerland; e Department of Business Economics, Health and Social Care, Research Methodology Competence Centre, University of Applied Sciences and Arts of Southern Switzerland, Manno, Switzerland; f Department of Urology, Ospedale F. Miulli, Acquaviva delle Fonti, Bari, Italy

**Keywords:** Bladder cancer, non-muscle invasive, intermediate and high risk, transurethral resection, electro-mediated drug administration, mitomycin C

## Abstract

**Objective:**

To evaluate the effectiveness of electro-mediated drug administration of mitomycin C (EMDA/MMC) after transurethral resection of the bladder tumour (TURBT) in preventing non-muscle-invasive bladder cancer (NMIBC) recurrence and progression and to explore clinical and demographic factors associated with treatment response.

**Patients and methods:**

Between April 2016 and August 2019, 112 patients diagnosed with intermediate- or high-risk NMIBC underwent a TURBT followed by an EMDA/MMC treatment. The percentage of treatment responders and progression-free survivors at 3 and 6 months were evaluated.

**Results:**

Follow-up data were available for 101 patients (90%) at 3 months and 92 (82%) at 6 months. Response rates to EMDA/MMC treatment were 85% at 3 months and 75% at 6 months, and progression-free rates were 94% and 90%, respectively. No statistically significant differences were seen between intermediate- and high-risk patients. A higher risk of tumour recurrence and progression was associated with previous Bacillus Calmette–Guérin (BCG) failure. According to the Clavien–Dindo classification, only low-grade complications were observed.

**Conclusions:**

EMDA/MMC after TURBT was associated with high response and progression-free rates at 3 and 6 months, with only low-grade adverse events. These results confirm the efficacy and safety of EMDA/MMC as a therapeutic option for both intermediate- and high-risk patients. However, patients with BCG failure responded poorly to EMDA/MMC.

**Abbreviations**: ACCI: age-adjusted Charlson Comorbidity Index; CHT: chemohyperthermia; CIS: carcinoma *in situ*; EMDA: electro-mediated drug administration; EORTC: European Organisation for Research and Treatment of Cancer; IQR: interquartile range; (N)MIBC: (non-)muscle-invasive bladder cancer; MMC: mitomycin C; OR, odds ratio; TURBT: transurethral resection of the bladder tumour

## Introduction

Non-muscle invasive bladder cancer (NMIBC) affects 70–80% of patients with bladder cancer (BC) [1–3]. NMIBC is the most common BC type, and a heterogeneous disease, characterised by a high risk of recurrence and a significant risk of progression to muscle-invasive disease after diagnosis. About seven patients diagnosed with NMIBC out of 10 experience a relapse, with muscle-invasive progression developing in ~15% of them [4,5].

Standard therapy for NMIBC consists of the complete transurethral resection of all visible bladder tumours (TURBT), usually followed by an intravesical treatment with immunotherapy (e.g. BCG) or chemotherapy [6]. However, standard therapy is related to high tumour recurrence and progression rates, implying lifelong intensive monitoring and large healthcare costs [7]. As NMIBC incidence has increased in several European countries [8] and there is a BCG shortage related to the increased global demand [9–11], it is essential to search for new more effective therapeutic options or strategies to improve the efficacy of existing drugs.

The search for more effective treatment options for NMIBC is currently ongoing, and several therapies (e.g. monoclonal antibodies, vaccines or gene therapy) are presently under investigation [12]. Intravesical drug administration is one of the most common treatment alternatives; current research on this therapy concentrate on improving the efficacy of intravesical delivery systems in terms of active drug transportation through the bladder wall [13]. During the last 20 years some devices have been introduced in clinical practice to improve the efficacy of passive absorption of the topically administered drugs, usually mitomycin C (MMC), mainly by increasing the bladder temperature (chemohyperthermia [CHT]) or through electro-mediated drug administration (EMDA), which improves the active transport of MMC [14,15]. Both therapeutic options will play an important role in the future management of NMIBC [14,16–22].

The present study aimed to assess the effectiveness of EMDA/MMC instillations after TURBT in preventing tumours recurrence and progression at 3 and 6 months in patients diagnosed with NMIBC, and to explore which clinical and demographic factors are associated with a favourable treatment response.

## Patients and methods

### Population and data collected

Patients were recruited between April 2016 and August 2019 in two medical centres located in Switzerland and Italy. The study was approved by the Local Ethics Committee (decision number: 2018–01470/CE3390), and all patients signed an informed consent.

All patients underwent an evaluation with abdominal CT scan before TURBT. Random biopsies of the healthy bladder mucosa were taken during every TURBT. Patients with T1 histological results underwent a second confirmatory TURBT within 1 month from the first one. Standard follow-up included evaluations after 3 and 6 months from TURBT, with a cystoscopy and a washing cytology. In presence of visible lesions, the patient also underwent a biopsy and eventually an additional TURBT. Patients intolerant to EMDA/MMC or reporting symptoms suggesting a disease relapse were evaluated before the 3 or 6 months deadlines.

Exclusion criteria for EMDA/MMC after TURBT (and therefore for participation in the study) were low-risk NMIBC (according to the guidelines of the European Organisation for Research and Treatment of Cancer, [EORTC] [5]), muscle-invasive bladder cancer (MIBC), non-transitional bladder cancer, upper urinary tract cancer, and known MMC intolerance. Patients recruited were both naïve and refractory or relapsing to BCG or MMC (not EMDA), diagnosed with intermediate- or high-risk NMIBC and treated with EMDA/MMC after a TURBT.

The treatment consisted of 30-min sessions of EMDA/MMC (Physion Mini 30N2, 20–23 mA, 40% [w/v] mitomycin dissolved in distilled water). Patients underwent up to eight EMDA/MMC weekly treatments (induction cycle) and 12 maintenance monthly sessions (maintenance cycle).

NMIBC recurrence was defined as a recurrence with a stage and grade of the tumour equal or lower than the diagnosis before treatment. NMIBC progression corresponded to a higher stage and/or grade of the recurrent non-muscle invasive tumour (<T2), while muscle-invasive progression was defined as a recurrence ≥T2.

We collected data on treatment response (defined as negative cystoscopy, cytology and/or histology at the corresponding time point) and progression-free survival (non-muscle-invasive or muscle-invasive progression) after 3 and 6 months. Patients’ demographics and clinical conditions data included gender, age, age-adjusted Charlson Comorbidity Index (ACCI), number of previous bladder cancer recurrences, previous treatments, EORTC risk classification at enrolment, and number of EMDA/MMC induction and maintenance sessions.

### Statistical analysis

Descriptive statistics are presented for the outcomes (proportions of treatment responders and progression-free survivors at 3 and 6 months), demographics, and clinical conditions. Categorical variables are described using percentages and continuous variables using medians and interquartile ranges (IQRs). Kaplan–Meier curves are used to present the evolution of treatment response at 3 and 6 months.

The associations between the main outcomes and the demographic and clinical variables were explored using the Fisher’s exact test for the relationships between categorical variables, and associations between categorical and continuous variables were assessed using the Mann–Whitney *U*-test, with *P* = 0.05 as the threshold for statistical significance. Odds ratios (ORs) were computed for statistically significant relationships between outcomes and binary (or dichotomised) variables.

No logistic regression analysis was carried out to evaluate the associations between the outcomes and the demographic and clinical variables, because the sample size was smaller than the minimal sample size necessary to obtain a robust model with reliable estimates [23].

All statistical analyses have been carried out with Stata/IC 16.0 (StataCorp, College Station, TX, USA).

## Results

### Patients’ disposition

**Figure 1.**
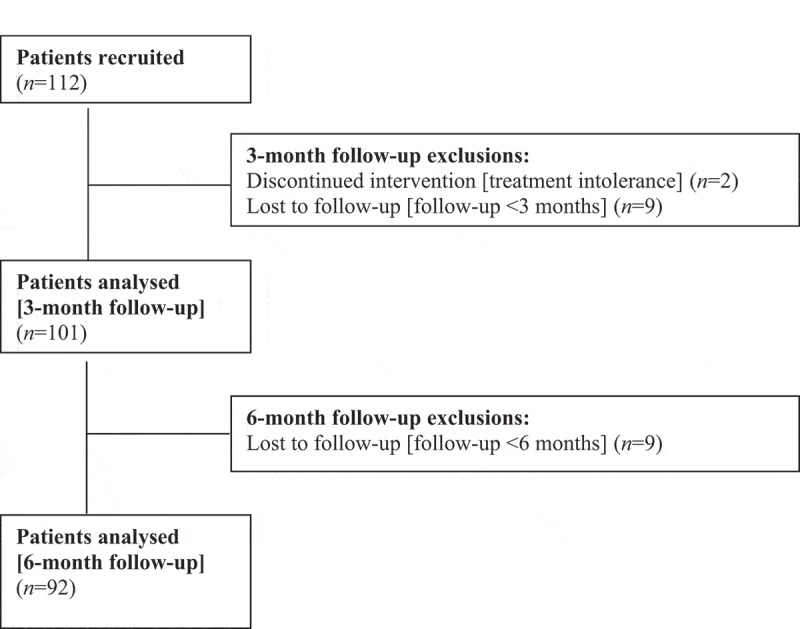
Patients’ disposition

Figure 1 shows the patients’ disposition. Between April 2016 and August 2019, 112 patients diagnosed with NMIBC underwent a complete TURBT followed by an EMDA/MMC treatment. In all, 96 patients were recruited in the Italian centre, while the remaining 16 were recruited in the Swiss centre. Two patients dropped out because of treatment intolerance at sessions 1 and 2, and nine had a follow-up of <3 months. Thus, 101 patients were included in the analysis after 3 months. Nine additional patients were lost to follow-up at 6 months, leaving 92 patients for the 6-months’ analysis.

### Descriptive statistics

Table 1 describes the patients’ demographics and clinical variables, as well as the outcomes after 3 and 6 months. Most patients were male (>90%), with an overall median age of 72 years. EORTC risk classification at the enrolment was intermediate for ~25% of the patients and high for the remaining 75%. The median ACCI score was 4. Slightly less than half of the patients (44%) had relapses prior to EMDA/MCC. Most patients (~75%) had no previous treatment, ~15% had been previously treated with MMC (but not EMDA), and slightly <10% were BCG refractory or relapsing (without previous EMDA treatments). Most patients (>85%) received eight instillations with EMDA/MMC and underwent at least one maintenance session. About 50% of the patients underwent six or more maintenance sessions.

**Table 1.** Patients’ demographics, clinical characteristics and outcomes

Variable	3 months (*n* = 101)	6 months (*n* = 92)
Gender, *n* (%) Woman Man	9 (8.9) 92 (91.1)	8 (8.7) 84 (91.3)
Age, years, median (IQR)	72 (14)	71 (14)
ACCI score, median (IQR)	4 (2)	4 (2)
Previous recurrence, *n* (%) No Yes	57 (56.4) 44 (43.6)	51 (55.4) 41 (44.6)
Previous treatments, *n* (%) None MMC (non-EMDA) BCG refractory BCG relapsing	78 (77.2) 14 (13.9) 4 (4.0) 5 (4.9)	70 (76.1) 14 (15.2) 3 (3.3) 5 (5.4)
EORTC risk classification, *n* (%) Intermediate risk High risk	27 (26.7) 74 (73.3)	25 (27.2) 67 (72.8)
EMDA treatment sessions, *n* (%) <8 instillations 8 instillations	13 (12.9) 88 (87.1)	12 (13.0) 80 (87.0)
Maintenance sessions, *n* (%) None 1–5 ≥6	14 (13.9) 36 (35.6) 51 (50.5)	13 (14.1) 29 (31.5) 50 (54.4)
Treatment response, *n* (%) Responder NMIBC recurrence NMIBC progression MIBC progression	86 (85.1) 9 (8.9) 4 (4.0) 2 (2.0)	69 (75.0) 14 (15.2) 4 (4.4) 5 (5.4)

At 3 months, 85.1% of the patients were responders and 94% were progression-free; 6% developed a progressive NMIBC or MIBC. The proportions of treatment responders and progression-free survivors were slightly lower at 6 months, but the difference was not statistically significant (*P* = 0.136).

**Figure 2.**
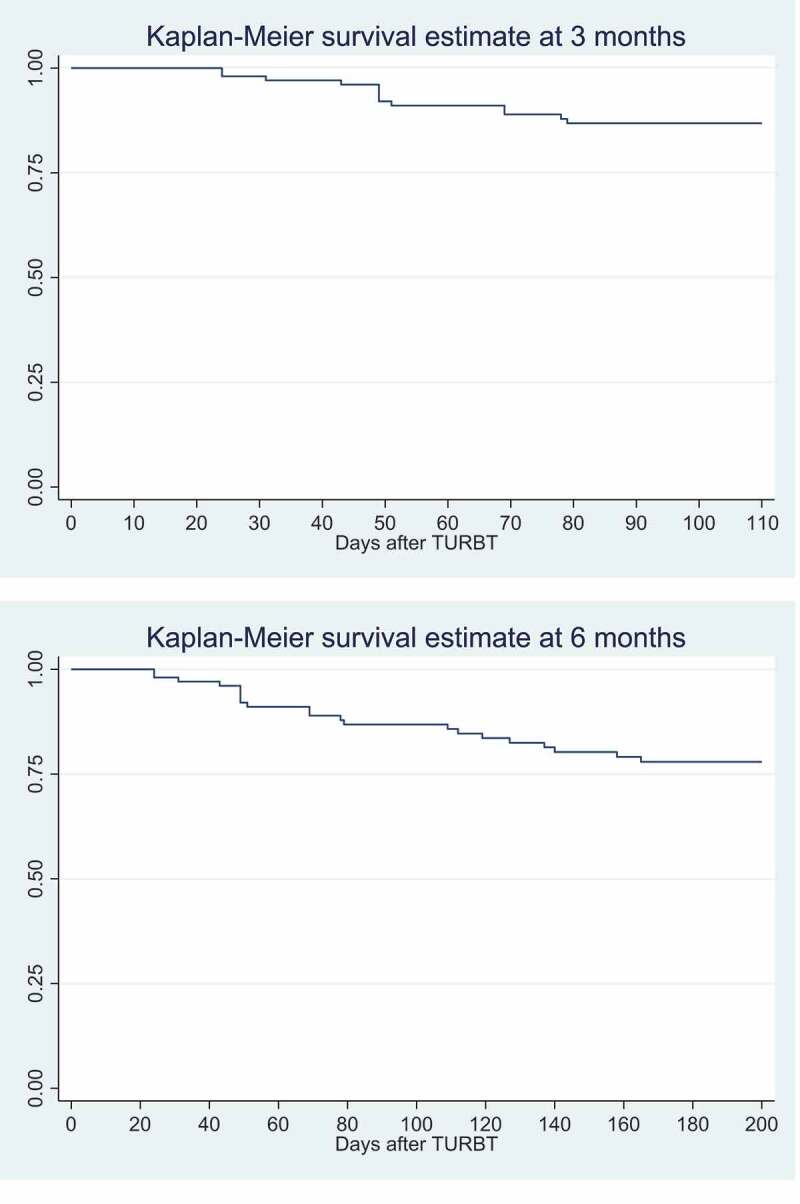
Kaplan–Meier survival estimates at 3 and 6 months

Figure 2 shows the Kaplan–Meier curves at 3 and 6 months. Both curves show that the first tumour recurrences appear within the first month after TURBT.

### Bivariate analysis

Gender, age, ACCI, the number of previous bladder cancer recurrences and EORTC risk classification at the enrolment were not significantly associated with treatment response or progression-free survival at 3 and 6 months. Both outcomes, however, were significantly related to previous treatments, number of EMDA/MMC instillation, and number of maintenance sessions (Table 2). Treatment responders were less likely to have had a previous BCG failure (3 months: OR 0.098, 95% CI 0.017–0.554; 6 months: OR 0.164, 95% CI 0.024–0.955), and they were less likely to have had less than eight EMDA treatment sessions (3 months: OR 0.133, 95% CI 0.031–0.607; 6 months: OR 0.179, 95% CI 0.040–0.768), and no maintenance sessions (3 months: OR 0.024, 95% CI 0.004–0.128; 6 months: OR 0.033, 95% CI 0.003–0.185). Similar results were obtained for progression-free survivors.

**Table 2.** Associations between treatment response/progression-free survival and previous BCG failure, number of EMDA/MMC instillations and number of maintenance sessions at 3 and 6 months

	Responder at 3 months (*n* = 101)		Responder at 6 months (*n* = 92)			Progression-free survivor at 3 months (*n* = 101)		Progression-free survivor at 6 months (*n* = 92)		
Variable	No	Yes	No	Yes	OR (95% CI)	No	Yes	No	Yes	OR (95% CI)
Previous BCG failure, *n* Yes No	5 10	4 82	5 18	3 66	3 months: 0.098 (0.017–0.554)** 6 months: 0.164 (0.024–0.955)*	3 3	6 89	3 6	5 78	3 months: 0.067 (0.008–0.647)** 6 months: 0.128 (0.019–0.942)*
<8 EMDA treatment sessions, *n* Yes No	6 9	7 79	7 16	5 64	3 months: 0.133 (0.031–0.607)** 6 months: 0.179 (0.040–0.768)**	4 2	9 86	4 5	8 75	3 months: 0.052 (0.004–0.446)** 6 months: 0.133 (0.024–0.841)*
No maintenance sessions, *n* Yes No	10 5	4 82	11 12	2 67	3 months: 0.024 (0.004–0.128)*** 6 months: 0.033 (0.003–0.185)***	5 1	9 86	5 4	8 75	3 months: 0.021 (0.001–0.232)*** 6 months: 0.085 (0.014–0.506)**

Fisher’s exact test statistical significance: *P < 0.05, **P < 0.01, ***P < 0.001.

### Safety

Complications were evaluated according to the Clavien–Dindo classification system [24,25]. EMDA/MMC administration was characterised by 10 low-grade adverse events: eight were Grade 1 complications (namely skin erythaema in six cases, catheter intolerance in two) and the remaining were Grade 2 (namely bladder tightness in one and bladder pain in one). Only two patients had to stop the treatment because of adverse events.

## Discussion

In the present study, EMDA/MMC proved to be a safe and effective treatment against the recurrence and progression of NMIBC after TURBT in a sample of intermediate- and high-risk patients [24]. Our present results confirm previous studies assessing the efficacy of EMDA/MMC for NMIBC treatment [18,26–30]. Several investigations have shown the efficacy of chemohyperthermia for the treatment of intermediate- and high-risk patients with NMIBC [31–33]. Our present results confirm and underline the efficacy of adjuvants device-assisted intravesical therapies for intermediate- and high-risk NMIBC management. These data justify more research to assess in comparative, prospective trials the clinical efficacy and cost-effectiveness of the various options [16].

Patients who are BCG refractory or relapsing or those with a fewer EMDA/MMC instillations and maintenance sessions were significantly associated with a reduced treatment response. The latter is probably related to the usual treatment/maintenance interruption for tumour relapse, which leads non-responders to have fewer sessions. Previous BCG failure represents a strong predictive factor of tumour recurrence/progression after TURBT followed by EMDA/MMC. Patients unresponsive to BCG represent a particular population with a high recurrence risk, for whom radical cystectomy remains the standard and most effective treatment [34–37]. Several treatment options for the management of these patients are under investigation, and preliminary results of studies using EMDA [38] and CHT [17,39,40] to prevent radical cystectomy have provided promising results. However, one of these studies also highlighted the lack of efficacy of chemotherapy for carcinoma *in situ* (CIS) [39]. This is consistent with the lack of efficacy of EMDA/MMC after TURBT for BCG unresponsive patients observed in our present study. Therefore, in these patients the same treatment may lead to different outcomes in terms of tumour recurrence and progression, and this is probably related to the large heterogeneity of the BCG unresponsive population [34–37]. BCG failures usually need careful and individualised therapies coupled with constant monitoring on a long-term perspective. One approach to this issue is to treat before BCG failure, by attempting to improve the efficacy of the first BCG treatment by combining it with other anti-cancer drugs. Two studies considered a sequential BCG-EMDA/MMC treatment and reported that this approach was superior to BCG therapy alone [41,42] even if tolerability was an issue [42]. Further research is needed to improve therapeutic efficacy before and/or after BCG failure.

In our present study, tumour recurrence and progression did not differ between patients with intermediate- and high-risk NMIBC. The intermediate-risk category is presently considered a grey zone between low- and high-risk NMIBC from a diagnostic and prognostic point of view and in terms of treatment modalities [43]. While therapeutic options for low- and high-risk patients are well established, there is no clear consensus on the optimal therapeutic protocol for intermediate-risk patients, a heterogeneous group characterised by a significantly varying risk of recurrence and progression [44].

### Limitations

The limits of the present study were its retrospective non-randomised nature and the short follow-up period. Moreover, at the moment of induction therapy with EMDA/MMC only three patients were diagnosed with CIS and no one had CIS associated to papillary tumours. Finally, no control group was available for the analysis.

## Conclusions

Our present study confirmed the clinical effectiveness and safety of the EMDA/MMC treatment after TURBT, with no difference in the outcomes between intermediate- and high-risk patients. The results also showed the particularly high propensity to tumour recurrence and progression for patients with a previous failure of BCG therapy.

Further studies are required to assess the clinical effectiveness of device-assisted intravesical therapies and therapeutic options after BCG failure. Moreover, additional research is needed for the revision and improvement of current risk classification methods and a better definition of treatment protocols for patients with intermediate-risk NMIBC.
